# *DFNA5* promoter methylation a marker for breast tumorigenesis

**DOI:** 10.18632/oncotarget.16654

**Published:** 2017-03-29

**Authors:** Lieselot Croes, Ken Op de Beeck, Patrick Pauwels, Wim Vanden Berghe, Marc Peeters, Erik Fransen, Guy Van Camp

**Affiliations:** ^1^ Center of Medical Genetics, University of Antwerp and Antwerp University Hospital, Edegem B-2650, Belgium; ^2^ Center for Oncological Research, University of Antwerp and Antwerp University Hospital, Edegem B-2650, Belgium; ^3^ Laboratory of Protein Chemistry, Proteomics and Epigenetic Signaling (PPES), Antwerp B-2610, Belgium; ^4^ StatUa Center for Statistics, University of Antwerp, Antwerp B-2000, Belgium

**Keywords:** DFNA5, breast cancer, DNA methylation, biomarker, detection

## Abstract

**Background:**

Identification of methylation markers that are sensitive and specific for breast cancer may improve early detection. We hypothesize that *DFNA5* promoter methylation can be a valuable epigenetic biomarker, based upon strong indications for its role as tumor suppressor gene and its function in regulated cell death.

**Results:**

Statistically different levels of methylation were seen, with always very low levels in healthy breast reduction samples, very high levels in part of the adenocarcinoma samples and slightly increased levels in part of the normal tissue samples adjacent the tumor. One of the CpGs (CpG4) showed the best differentiation. A ROC curve for *DFNA5* CpG4 methylation showed a sensitivity of 61.8% for the detection of breast cancer with a specificity of 100%.

**Materials and Methods:**

We performed methylation analysis on four CpGs in the *DFNA5* promoter region by bisulfite pyrosequencing on 123 primary breast adenocarcinomas and 24 healthy breast reductions. For 16 primary tumors, corresponding histological normal tissue adjacent to the tumor was available.

**Conclusions:**

We conclude that *DFNA5* methylation shows strong potential as a biomarker for detection of breast cancer. Slightly increased methylation in histologically normal breast tissue surrounding the tumor suggests that it may be a good early detection marker.

## INTRODUCTION

The *deafness, autosomal dominant 5* (*DFNA5*) gene was identified in our lab in 1998, as a gene causing autosomal dominant non-syndromic hearing loss [1]. Experiments in our laboratory have demonstrated that the DFNA5 protein has the capacity to induce regulated cell death [2–4]. In addition, we recently showed that the mitochondria and especially the MAPK-related pathways play a role in DFNA5-induced regulated cell death [2, 3]. A number of papers on DFNA5 have been published, pointing towards a possible involvement in cancer [2–14]. Furthermore, *DFNA5* has been identified in several tumor suppressor genomic methylation screens [8, 9, 11].

Methylation of promoter CpG islands, frequently associated with transcriptional silencing, may serve as a mechanism to inactivate tumor suppressor genes in cancer [15–19]. This is also true for breast cancer [16–19]. Thus, the identification of methylation markers that are sensitive and specific for breast cancer may improve early detection, which is of tremendous importance in achieving a better prognosis [20, 21].

Epigenetic silencing through *DFNA5* methylation was previously shown in 52% of primary gastric tumors [11] and in respectively 65% [9] and 34% [6] of colorectal cancers. In 2008, Kim et al. performed a *DFNA5* methylation analysis in breast cancer on a limited number of samples (*N* = 34) using a PCR-based methylation assay, analyzing only a single CpG site [10]. They showed *DFNA5* methylation in 53% of primary breast cancer samples and 15.3% of 13 histological normal breast tissues at a distance of the tumor. In seven breast samples of healthy women, *DFNA5* methylation was completely absent. The *DFNA5* methylation status correlated positively with lymph node metastasis [10]. However, the number of samples and the associations with clinicopathological and survival parameters in the latter study were limited.

In this study, we aimed to analyze the methylation status of *DFNA5* in breast cancer in more detail, potentially resulting in the identification of a new detection and/or prognostic marker. We hypothesize that *DFNA5* promoter methylation can be a valuable epigenetic biomarker, based upon strong indications for its role as tumor suppressor gene and its function in regulated cell death. We analyzed *DFNA5* promoter methylation in breast adenocarcinomas, normal breast tissues (matched tumor) and healthy breast reduction tissues in a large number of samples (*N* = 123). We also looked for associations between *DFNA5* methylation and clinicopathological and survival parameters.

## RESULTS

### *DFNA5* promoter methylation in primary untreated breast adenocarcinomas compared to healthy controls

*DFNA5* promoter methylation was investigated in 123 primary breast adenocarcinoma samples and 24 healthy breast reduction samples. All analyses were corrected for age because of the age difference between both groups (age range: 28–89 years for the breast adenocarcinomas and 17–69 years for the healthy breast reductions). Our analysis showed a statistically significant difference in the average *DFNA5* promoter methylation percentage between the primary breast adenocarcinomas and the healthy breast reductions (*p* = 1.6*10^−3^; R^2^ adjusted = 0.160). The median *DFNA5* promoter methylation was 9.00% [range: 1.25%–86.75%] for the breast adenocarcinomas, compared to 3.75% [range: 1.50%–7.00%] for the healthy breast reductions. To assess the reproducibility of our results, we calculated the intraclass correlation coefficient (ICC) for each of the two techniques used. When repeating the analysis starting from pyrosequencing, an ICC of 0.998 [95% CI: 0.997–0.999] was obtained, whereas an ICC of 0.924 [95% CI: 0.858–0.960] was observed when repeating the analysis starting from PCR.

**Figure 1:**
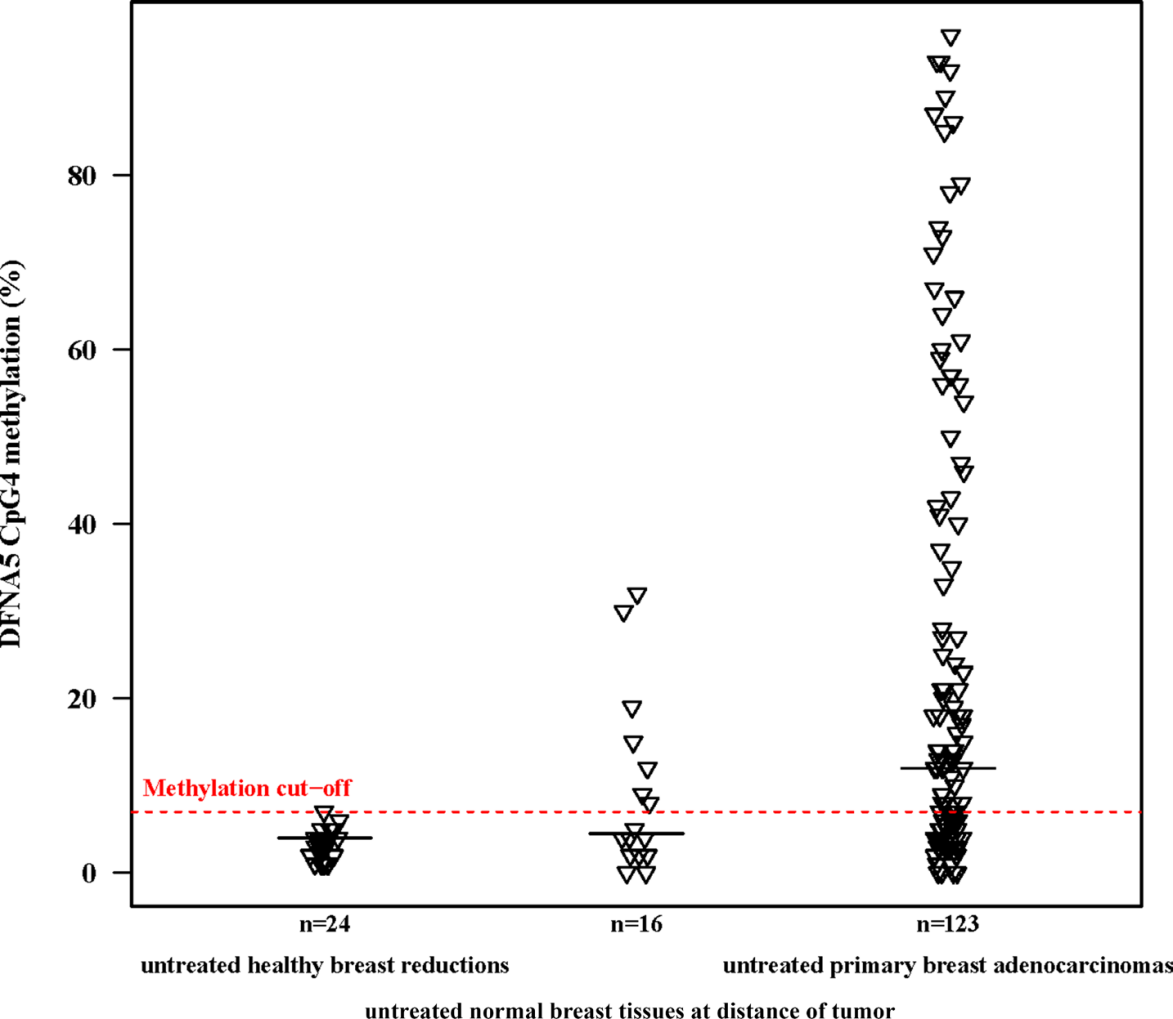
*DFNA5* CpG4 methylation percentages of primary breast adenocarcinomas compared to normal breast tissues at a distance of the tumor and healthy breast reductions The plot shows *DFNA5* CpG4 promoter methylation percentages in 123 untreated, primary breast adenocarcinomas; 16 untreated, histological normal breast tissues at a distance of the tumor and 24 untreated, healthy breast reduction samples of non-cancerous patients. The optimal methylation cutoff (7.0%), determined by a ROC analysis, showed *DFNA5* CpG4 methylation in 61.8% of 123 breast adenocarcinomas, in 43.8% of 16 histological normal breast tissues at a distance of the tumor and in none of 24 healthy breast reduction samples. The horizontal black lines indicate the median *DFNA5* CpG4 methylation percentage per group.

Based on the R^2^-value of the linear regression model, we determined which of the 4 individual CpGs differentiated best between breast adenocarcinoma samples and healthy breast reduction samples. A statistically significant difference in methylation between both groups was observed when looking at CpG1 (*p* = 0.017; R^2^ adjusted = 0.096), CpG3 (*p* = 7.5*10^−3^; R^2^ adjusted = 0.148) or CpG4 alone (*p* = 6.1*10^−4^; R^2^ adjusted = 0.168; Figure 1) alone. Based on the *p*-value and the R^2^-value, the methylation percentage of CpG4 was better suited to differentiate between breast adenocarcinomas and healthy controls than the average *DFNA5* methylation percentage. Therefore, *DFNA5* CpG4 methylation percentage was used in the subsequent analyses. All analyses were also performed with the average *DFNA5* methylation over all four CpGs and this did not affect the overall conclusions (data not shown in this manuscript). The median *DFNA5* CpG4 methylation percentage for the breast adenocarcinomas was 12% [range: 0%–96%], compared to 4% [range: 1%–7%] for the healthy breast reductions (Figure 1). Methylation values of CpG2 were least suited to differentiate between breast cancer and healthy control samples (*p* = 0.053; R^2^ adjusted = 0.073).

**Figure 2:**
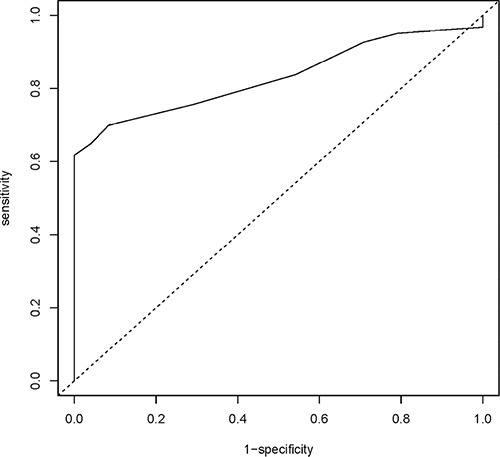
*DFNA5* CpG4 methylation percentage as a biomarker for breast adenocarcinomas Sensitivity and specificity at various cutoff values for our dataset (123 breast adenocarcinomas and 24 healthy breast reductions) are shown in the ROC curve. The full line represents the ROC curve. The dotted line represents the line of no discrimination between tumor and healthy breast samples. The determined optimal cutoff value for *DFNA5* CpG4 methylation is 7.0%.

Based upon the *DFNA5* CpG4 methylation percentages of breast adenocarcinomas and healthy breast reductions, we constructed a receiver operating characteristic (ROC) curve. The area under the curve (AUC) was 0.830 [95% CI: 0.765–0.896] (Figure 2). The optimal sensitivity and specificity for the detection of breast cancer was obtained with a methylation cutoff of 7.0%. *DFNA5* CpG4 methylation was considered positive if the methylation percentage was higher than the cutoff value. We detected *DFNA5* CpG4 methylation in 76 out of 123 breast cancer patients, yielding a sensitivity of 61.8% for the detection of breast cancer. Moreover, none of the 24 healthy controls tested positive for *DFNA5* CpG4 methylation, which resulted in a specificity of the assay of 100% in our dataset.

### *DFNA5* promoter methylation in primary untreated breast adenocarcinomas and histologically normal breast tissues at a distance of the tumor

**Figure 3:**
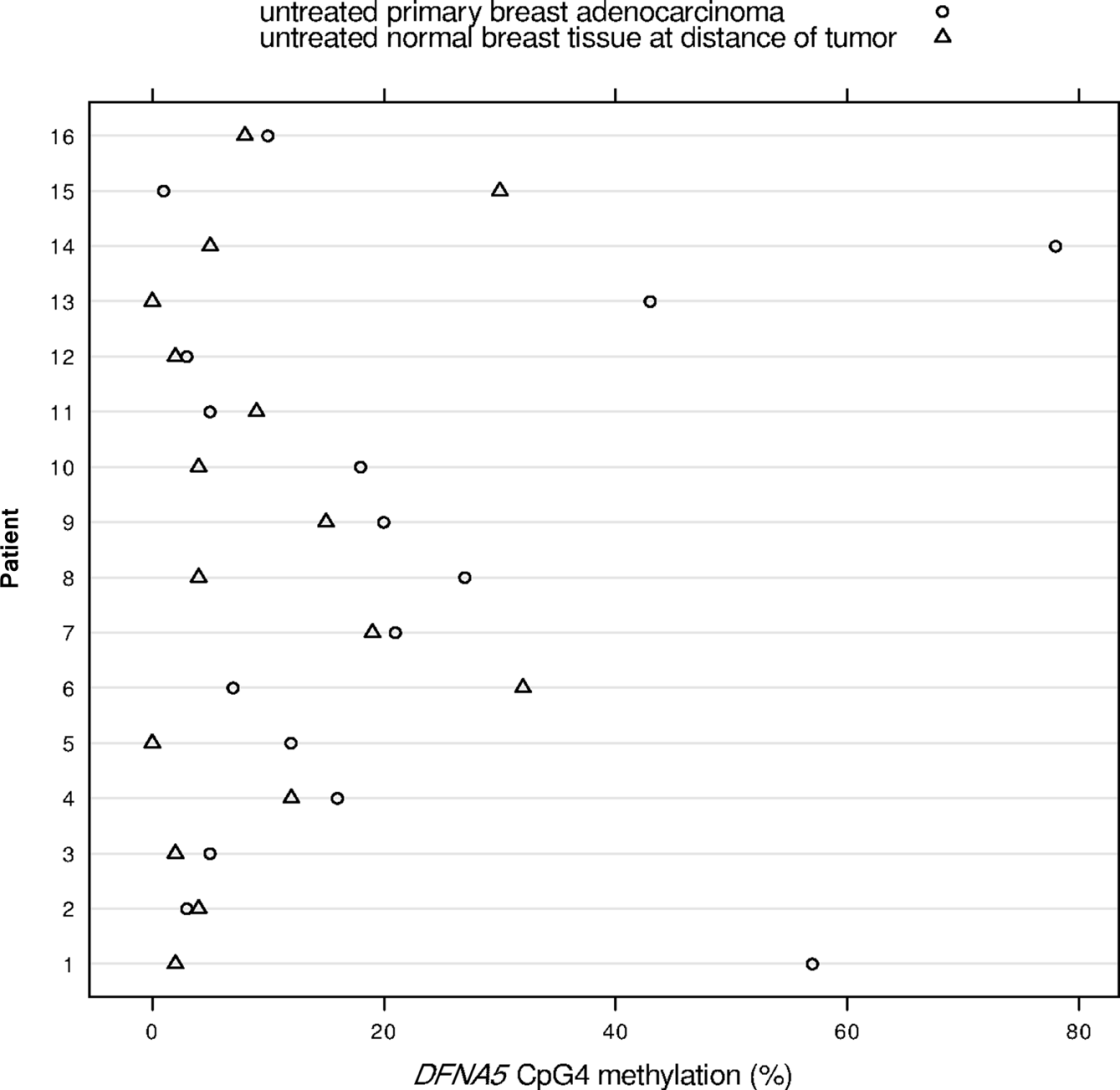
*DFNA5* CpG4 methylation percentages in 16 paired breast adenocarcinomas and histologically normal breast tissues at a distance of the tumor The x-axis shows the *DFNA5* CpG4 methylation percentage and each number on the y-axis depicts a patient, from whom both breast adenocarcinoma tissue and histologically normal breast tissue at a distance of the tumor were available.

We had access to tissues of 16 breast cancer patients from whom both breast adenocarcinoma tissue and histologically normal breast tissue at a distance of the tumor were available. The *DFNA5* methylation values obtained after pyrosequencing were analyzed using the paired samples *t*-test (Figure 1). We were not able to find a significant mean difference in *DFNA5* CpG4 methylation between breast cancer and matched normal samples (*p* = 0.10; Figure 3). We observed a median *DFNA5* CpG4 methylation difference of 3.5% [range: -29%–73%] between breast cancer and matched normal samples. Figure 3 reveals that in 75% (12/16) of the patients the *DFNA5* CpG4 methylation percentage was higher in the tumor sample compared to the normal sample. Interestingly, in 25% (4/16) of the patients, the *DFNA5* CpG4 methylation percentage in the matched normal sample was higher compared to the tumor sample.

### Associations between *DFNA5* CpG4 methylation and clinicopathological parameters

Association analyses between *DFNA5* CpG4 methylation percentage and available clinicopathological parameters were performed (Table 1). Not all clinicopathological parameters were available for all patients. A statistically significant association between *DFNA5* CpG4 methylation percentage and *human epidermal growth factor receptor 2* (*HER2*) amplification in 58 breast adenocarcinoma patients (45 without *HER2* amplification and 13 with *HER2* amplification) was found (*p* = 0.030; Table 1). The median *DFNA5* CpG4 methylation was 19% [Q1-Q3: 13%–43%] for the breast adenocarcinomas with *HER2* amplification, compared to 8% [Q1-Q3: 4%–18%] for the breast adenocarcinomas without *HER2* amplification. No associations were found between *DFNA5* CpG4 methylation and ischemia time, pathological tumor-node-metastasis (pTNM) staging, estrogen receptor (ER) status, progesterone receptor (PgR) status, lymphovascular invasion, tumor grade (Nottingham grading system), mitotic activity index (MAI) or maximal tumor diameter (Table 1).

**Table 1:** Clinicopathological characteristics of the 123 breast adenocarcinomas

Clinicopathological parameter	Number (%)	*P*-value
**pT (pTNM)**		0.25
1	54 (43.9)	
2	35 (28.5)	
3	5 (4.1)	
4	3 (2.4)	
unknown	26 (21.1)	
**pN (pTNM)**		0.80
0	56 (45.5)	
1	34 (27.7)	
2	3 (2.4)	
3	1 (0.8)	
unknown	29 (23.6)	
**ER**		0.20
ER+	100 (81.3)	
ER-	19 (15.4)	
unknown	4 (3.3)	
**PgR**		0.17
PgR+	78 (63.4)	
PgR-	36 (29.3)	
unknown	9 (7.3)	
***HER2***		0.030
*HER2+*	13 (10.6)	
*HER2-*	45 (36.6)	
unknown	65 (52.8)	
**Lymphovascular invasion**		0.81
yes	22 (17.9)	
no	27 (22.0)	
unknown	74 (60.2)	
**Tumor grade**		0.13
low grade	42 (34.1)	
intermediate grade	29 (23.6)	
high grade	30 (24.4)	
unknown	22 (17.9)	
**MAI**		0.64
**Tumor diameter**		0.32

*P*-values of the associations between *DFNA5* CpG4 methylation and the clinicopathological parameters are indicated in the third column. Some clinicopathological parameters were not available for all patients.

### Effect of *DFNA5* CpG4 methylation on survival

Overall survival (OS), progression free survival (PFS) and disease free survival (DFS) were investigated by survival analysis over a 5-year period. Kaplan Meier and Cox proportional hazard analyses were performed to determine the prognostic value of *DFNA5* CpG4 methylation in breast adenocarcinoma. Follow-up data were not available for all patients.

**Figure 4:**
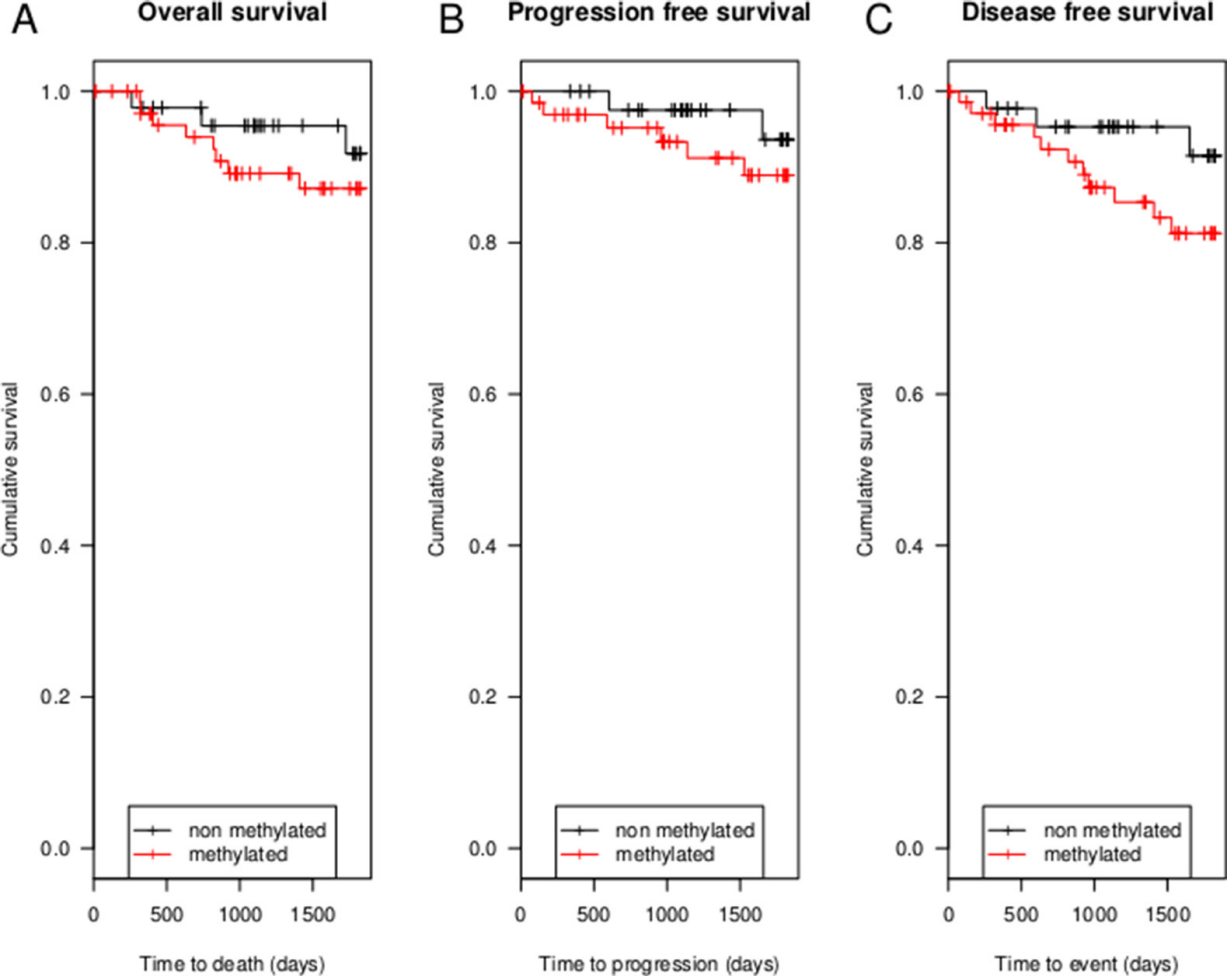
Kaplan Meier analyses of 5-year OS, 5-year PFS and 5-year DFS (**A**) shows 5-year OS in 120 breast adenocarcinoma patients, 11 patients died in 5 years after diagnosis. In (**B**) 5-year PFS in 111 breast adenocarcinoma patients is shown. In only 8 patients recurrence or metastatic disease occurred. (**C**) shows 5-year DFS in 116 patients. In 5 years after diagnosis a total of 14 patients have had metastatic disease, recurrence or died.

Kaplan Meier analyses were performed, comparing 2 groups based upon the dichotomized *DFNA5* CpG4 methylation values, using the cutoff based on the ROC analysis. In our dataset (*N* = 120), with a limited number of events (*N* = 11), there was no statistically significant difference in 5-year OS between methylated (*N* = 47) and non-methylated breast adenocarcinomas (*N* = 73) (*p* = 0.39; Figure 4). The 5-year OS rate was 89.0% (65/73) for the methylated and 93.6% (44/47) for the non-methylated breast adenocarcinoma patients. For the 5-year PFS, the number of events was also limited (*N* = 8) in our dataset (*N* = 111). The 5-year PFS rate was 91.0% (61/67) for the methylated and 95.5% (42/44) for the non-methylated breast adenocarcinoma patients (*p* = 0.36; Figure 4). The 5-year DFS rate was 84.5% (60/71) for the methylated and 93.3% (42/45) for the non-methylated breast adenocarcinoma patients, with a total of 14 events in 116 patients (*p* = 0.16; Figure 4; Supplementary Table 1).

Cox proportional hazard models were fit to estimate the effect of the non-dichotomized *DFNA5* CpG4 methylation percentages, accounting for age. The trend towards association between *DFNA5* CpG4 methylation and 5-year DFS could be confirmed (*p* = 0.051; Supplementary Table 1). *DFNA5* CpG4 methylation percentage had no statistically significant effect on neither 5-year OS (*p* = 0.19), nor on 5-year PFS (*p* = 0.10).

The impact of other covariates, including radiotherapy, adjuvant chemotherapy, adjuvant trastuzumab and hormonal therapy on 5-year OS, PFS and DFS could not be tested due to the limited number of events in our dataset.

## DISCUSSION

In this study we evaluated the potential use of *DFNA5* promoter methylation as a biomarker for the detection of breast cancer. We analyzed four specific CpGs in the promoter of *DFNA5* (Supplementary Figure 1). These CpG dinucleotides were of interest because Akino et al. [11] found that methylation of the region around the transcription start site of *DFNA5* correlated with silencing of the gene. As we demonstrated that the methylation percentage of CpG4 alone was better suited to differentiate between breast adenocarcinomas and healthy controls than the average *DFNA5* methylation percentage, we performed all our analyses using *DFNA5* CpG4 methylation percentages. The median *DFNA5* CpG4 methylation percentage was significantly higher (12%) in primary breast adenocarcinomas compared to healthy breast reduction samples (4%) (Figure 1). These results were used to construct a ROC curve that had an AUC of 0.830 (Figure 2). Based on this ROC curve, we were able to determine a methylation cutoff (7%), enabling us to dichotomize our study population. Using this cutoff, *DFNA5* CpG4 was methylated in 61.8% of the 123 primary breast adenocarcinomas and in none of the 24 breast samples from healthy women without cancer undergoing a breast reduction. We conclude that *DFNA5* CpG4 methylation can be a biomarker for the detection of breast cancer in solid biopsies. Although a different CpG in the *DFNA5* promoter was analyzed and a different technique (TaqMan-MSP) was used, our results are in line with those obtained by Kim et al. in 2008. They demonstrated *DFNA5* methylation in 53% (18/34) of the breast adenocarcinomas, 15.4% (2/13) of the normal breast tissues at a distance of the tumor and none (0/7) of the normal breast tissues from non-cancerous patients [10].

Analysis of *DFNA5* CpG4 methylation in histologically normal breast tissues at a distance of the tumor showed a higher *DFNA5* CpG4 methylation percentage in 25% (4/16) of the matched normal samples compared to the tumor samples (Figure 3). These observations can be explained by “field cancerization”, which is the occurrence of genetic, epigenetic and biochemical aberrations in structurally intact cells in histologically normal tissues adjacent to cancerous lesions. The concept of field cancerization was first described in oral squamous cell carcinoma by Slaughter et al. in 1953 [22]. Since then, field cancerization has been described in many different organ systems, including breast cancer [23–27]. In addition to genetic abnormalities (such as chromosomal anomalies and loss of heterozygosity) epigenetic alterations, in particular changes in the DNA methylation status, have been found in normal-appearing tissue surrounding the tumor site in breast cancer [24, 28–33]. Moreover, epigenetic modifications are believed to be early events in breast cancer development due to their presence in pre-invasive ductal carcinoma *in situ* (DCIS) [32, 34–36], which makes them very suitable as early detection biomarkers. We do not know whether *DFNA5* methylation is an early event as currently *DFNA5* methylation has not been analyzed in DCIS. However, our data and the data from Kim et al. [10] suggest that *DFNA5* methylation might be an early biomarker, making it a suitable candidate to include in a panel of breast cancer detection markers. Further research on the role of *DFNA5* in field cancerization is needed, as this phenomenon is regarded as clinically significant due to its presumed role in the local recurrence of cancer. Indeed, the region showing these molecular abnormalities is not always completely removed by surgery and therefore might lead to newly occuring neoplasms.

*DFNA5* CpG4 methylation performs well compared to other gene promoter methylation markers described in literature thus far. However, combining *DFNA5* CpG4 methylation with other markers could improve its sensitivity for the detection of breast cancer, as has been observed for other methylation markers. One example is the *Ras-associated domain family member 1* (*RASSF1A)* gene, one of the most frequently hypermethylated genes in human cancer. CpG island hypermethylation of *RASSF1A* has been demonstrated in 49%–77% of breast cancers [33, 37–39]. In a comprehensive study of *RASSF1A* promoter methylation in breast tissue samples, it was demonstrated that primary tumors had significantly higher promoter methylation compared to healthy breast reduction samples, while normal breast samples at a distance of the tumor had intermediate methylation levels [24]. These results correspond to our findings in *DFNA5*.

A next step in the study of the potential utility of *DFNA5* CpG4 methylation as a biomarker, is to analyze *DFNA5* CpG4 methylation in circulating DNA from both breast cancer patients and healthy individuals. Several studies have provided proof of principle for the detection of promoter hypermethylation of breast adenocarcinoma derived DNA in blood [40–42]. Using liquid biopsies, *DFNA5* CpG4 methylation has the potential to be a suitable low invasive early detection biomarker for breast cancer.

We found that *DFNA5* CpG4 methylation was associated with *HER2* amplification (Table 1). Currently, we do not have an explanation for this observation. Further functional studies are needed to elucidate the cause of the association with *HER2* status, possibly leading to more insights into tumorigenesis, or a better molecular subclassification. Like Kim et al. [10], we could not find an association with ER status in our study population, which is in contrast to the study of Thompson and Weigel [14]. A possible explanation could be that in the study of Thompson and Weigel *DFNA5* expression in cell lines and 29 primary breast tumors was analyzed instead of *DFNA5* methylation [14]. Moreover, the scoring system for ER status has changed over time [43, 44]. Furthermore, we were not able to find an association with lymph node metastasis (pN), which is in contrast to the study of Kim et al. [10].

The survival models for OS, PFS and DFS were not statistically significant, possibly due to the limited number of events in our study population. However, the survival curves suggest a trend, wherein breast cancer patients without *DFNA5* CpG4 methylation perform better compared to breast cancer patients with *DFNA5* CpG4 methylation (Figure 4). The strongest trend is observed for DFS. Larger prospective studies are needed to investigate the prognostic role of *DFNA5* methylation in breast cancer.

Finally, *DFNA5* has never been identified in classic tumor suppressor gene screens that aim to identify genes with inactivating somatic mutations. Moreover, we consulted the COSMIC database and compared the number of somatic *DFNA5* mutations with somatic mutations in non-cancer genes, similar results were found (data not shown in this manuscript). Taken together, we can conclude that inactivation of *DFNA5* through gene mutations, in contrast to promoter hypermethylation, is not a main mechanism for *DFNA5* in cancer.

In conclusion, we demonstrated that 1) *DFNA5* CpG4 methylation can be a biomarker for the detection of breast cancer in solid biopsies, 2) *DFNA5* is methylated in breast adenocarcinomas in contrast to healthy breast reduction samples, 3) *DFNA5* methylation is often present in normal breast tissue surrounding the tumor, which may be explained by field cancerization. Firstly, this suggests that *DFNA5* methylation is an early event, which offers opportunities for *DFNA5* methylation as early detection maker for breast cancer. Secondly, this indicates the importance of using healthy reference tissue from non-cancerous patients in detection biomarker research. Further research is needed to investigate if *DFNA5* methylation could reliably be analyzed in liquid biopsies, so that it may be developed as low invasive, early detection biomarker for breast cancer.

## MATERIALS AND METHODS

### Study population and tissue samples

We collected 123 well-characterized primary breast adenocarcinomas (108 ductal– 10 lobular– 2 mixed– 3 unknown) and 24 breast samples from women without cancer undergoing a breast reduction. If available, we also retrieved histologically normal breast tissue from breast cancer resection specimens (*N* = 16; 14 ductal– 2 mixed). All samples were formalin-fixed, paraffin-embedded (FFPE) and were retrieved from the tumorbank of the Antwerp University Hospital (tumorbank@UZA). Characteristics of the study population are shown in Table 1. This study was approved by the ethical committee of the Antwerp University Hospital and the University of Antwerp.

### DNA isolation

A 5μm-section of each tissue block was stained with hematoxylin and eosin (HE) and inspected by the study pathologist (Prof. Dr. P. Pauwels). Inclusion criteria for tumor samples were primary malignancy without necrosis and tumor cell content of at least 40%. Ten sections (5 μm thick) were deparaffinized and subjected to DNA extraction using the QIAamp^®^ DNA FFPE Tissue Kit (Qiagen, Hilden, Germany) according to the manufacturer's instructions.

### Bisulfite conversion, PCR and pyrosequencing

Bisulfite modification was performed using 2 μg genomic DNA as input in a MethylEasy™ Xceed Kit (Human Genetic Signatures, Sydney NSW 2113, Australia) according to the manufacturer's instructions. For PCR, bisulfite-modified DNA was amplified by AmpliTaq Gold^®^ DNA Polymerase (Applied Biosystems, California 94404, USA) using the following degenerate primers (5′-Biotin-RAACCCCTCCCRCAACCT-3′ and 5′-GGYGGAGAGAGGGTTYGTT-3′, Y = C/T and R = A/G; Supplementary Figure 1). The PCR program consisted of an initial enzyme activation at 95°C for 10 minutes, followed by 50 cycles of 45 seconds at 95°C, 45 seconds at 60°C and 45 seconds at 72°C; and a final extension at 72°C for 10 minutes. Subsequently, four specific CpG dinucleotides around the transcription start site of *DFNA5* were sequenced using the following sequencing primer (5′-YGGGYGTTTTAGAGT-3′, Y = C/T) on the PyroMark Q24 platform (Qiagen, Hilden, Germany) according to the manufacturer's instructions (Supplementary Figure 1). Analysis of the results was performed with the PyroMark Q24 software (Qiagen, Hilden, Germany).

### Statistical analysis

All analyses were performed with log10 transformed methylation percentages to obtain normality. Due to the age difference between the healthy samples and the breast adenocarcinoma samples, age was included as a covariate throughout all analyses. For the comparison of *DFNA5* methylation percentages between breast adenocarcinomas and healthy breast reductions, linear regression models were fit with the logarithm of the methylation percentage as dependent variable and disease status as independent variable. Intraclass correlation was calculated for absolute agreement. The association between *DFNA5* CpG4 methylation percentage and clinicopathological parameters was tested by fitting linear regression models with the logarithm of the methylation percentage as dependent variable and the clinicopathological parameter as independent variable. The diameter, MAI and ischemia time were entered as independent variables after log10 transformation. To analyze whether ischemia time, the time between resection of the tissue and the time of fixation in formalin, had an influence on *DFNA5* methylation, we corrected for disease status. For survival analyses, patients were censored at time of last follow-up, with a maximum of 5 years. OS was defined as: the time of diagnosis to death; PFS as: the time of diagnosis to recurrence or metastatic disease and DFS as: the time of diagnosis to recurrence, metastatic disease or death. All *p*-values are two-sided, and *p*-values less than or equal to 0.05 were considered statistically significant. Data analysis was performed using SPSS software (version 22; SPSS Inc., Chicago, IL).

## SUPPLEMENTARY MATERIALS FIGURES AND TABLES



## Abbreviations

*DFNA5*: deafness, autosomal dominant 5
ICC: intraclass correlation coefficient
ROC: receiver operating characteristic
AUC: area under the curve
*HER2*: human epidermal growth factor receptor 2
pTNM: pathological tumor-node-metastasis
ER: estrogen receptor
PgR: progesterone receptor
MAI: mitotic activity index
OS: overall survival; PFS: progression free survival
DFS: disease free survival
DCIS: ductal carcinoma *in situ*
*RASSF1A*: Ras-associated domain family member 1
