# Supplementary material for: DFNA5 promoter methylation a marker for breast tumorigenesis
Source: Oncotarget. 2017 Mar 29;8(19):31948–58. doi: 10.18632/oncotarget.16654 (PMC5458261; doi:10.18632/oncotarget.16654)
Supplement: Supplementary file 1 [file oncotarget-08-31948-s001.pdf]

DFNA5 promoter methylation - a marker for breast tumorigenesis

Supplementary Materials

Supplementary Table 1: 5-year DFS

		DFNA5 CpG4 methylation (%)	Event	Days to event	Age at diagnosis (years)
**Non-methylated breast adenocarcinomas**					
	1	5	death	260	47
	2	6	metastasis	1652	56
	3	3	metastasis	603	44
**Methylated breast adenocarcinomas**					
	1	15	metastasis	1528	71
	2	24	death	632	73
	3	12	death	321	55
	4	12	death	1408	55
	5	61	death	923	50
	6	57	metastasis	959	42
	7	92	death	820	70
	8	60	metastasis	589	80
	9	73	recurrence	1138	68
	10	18	metastasis	154	54
	11	86	metastasis	75	53

The table shows events that happened in the methylated and non-methylated breast adenocarcinoma patients within 5 years, with *DFNA5* CpG4 methylation (%), event, days to event and the age at diagnosis of the breast adenocarcinoma patients. Only the event that has happened first was reported.

5'–**GCA**GCCCACTCTTCCCGAGAGGCCCCGACATCTCCCGGGTGAGTCCCGAAAAGCGCGCGGGGACTCCGGCTGAGC

GCTGGGCCGTCAGCAACCCGGCTCCCAGAGAGTCACACGAAGGAGGGGAAGCGGCTCTCTCTGGGGCTTCTGGGAG

B

GTCTGGCCC**GGGCCCCTCCCGCAGCCTC**C**CG_4**G**CG_3**GCCAGTCC**$CG_2$$CG_1$GCTCTGGGGCGCCCG**GACCGAGCAAA

GGTCCCGGCG**GGCGGGCCCTCTCTCCGCC**CTCCCCAAGCGCCCGCCCCCCGCCGGC–3'

Supplementary Figure 1: Location of the four analyzed CpG dinucleotides in the *DFNA5* genomic sequence. The sequence indicated in light grey is exon 1. The sequence indicated in dark grey is the intronic sequence between exon 1 and 2. The four specific CpG dinucleotides, analyzed in this study, are located in the intron between exon 1 and exon 2 and are indicated in red. GCA: transcription start site, located in exon 1. B→: biotinylated forward primer (5′-Biotin-RAACCCCTCCCRCAACCT-3′), ←: reverse primer (5′-GGYGGAGAGAGGGTTYGTT-3′), ←: sequencing primer (5′-YGGGYGTTTTAGAGT-3′).
